# Wild Radish (*Raphanus raphanistrum* L.) Is a Potential Reservoir Host of Cucurbit Chlorotic Yellows Virus

**DOI:** 10.3390/v14030593

**Published:** 2022-03-13

**Authors:** Saritha R. Kavalappara, David G. Riley, Paulo S. G. Cremonez, Jermaine D. Perier, Sudeep Bag

**Affiliations:** 1 Department of Plant Pathology, University of Georgia, Tifton, GA 31793, USA; 2 Department of Entomology, University of Georgia, Tifton, GA 31793, USA; dgr@uga.edu (D.G.R.); paulogimz@uga.edu (P.S.G.C.); jermaine.perier@uga.edu (J.D.P.)

**Keywords:** crinivirus, cucurbit chlorotic yellows virus, wild radish, whitefly

## Abstract

Cucurbit chlorotic yellows virus (CCYV) belongs to the genus *Crinivirus* and is part of a complex of whitefly-transmitted viruses that cause yellowing disease in cucurbits. In the southeastern USA, heavy incidences of CCYV have been observed on all cucurbits grown in the fall. CCYV was detected from wild radish (*Raphanus raphanistrum* L.), a common weed that grows in the southeastern USA by high-throughput sequencing as well as RT-PCR. CCYV sequence from wild radish was 99.90% and 99.95%, identical to RNA 1 and RNA 2 of cucurbit isolates of CCYV from the region. Transmission assays using whiteflies demonstrated that wild radish is a good host for CCYV. Whiteflies were also able to acquire CCYV from wild radish and transmit the virus to cucurbit hosts, which developed typical symptoms associated with CCYV. Using quantitative PCR, the titer of CCYV in wild radish was also estimated to be on par with that of cucurbit hosts of the virus. Whitefly bioassays revealed that wild radish is an acceptable feeding and reproductive host plant. These results indicate that wild radish could serve as a reservoir host for CCYV in the USA and other parts of the world where similar conditions exist.

## 1. Introduction

**Figure 1:**
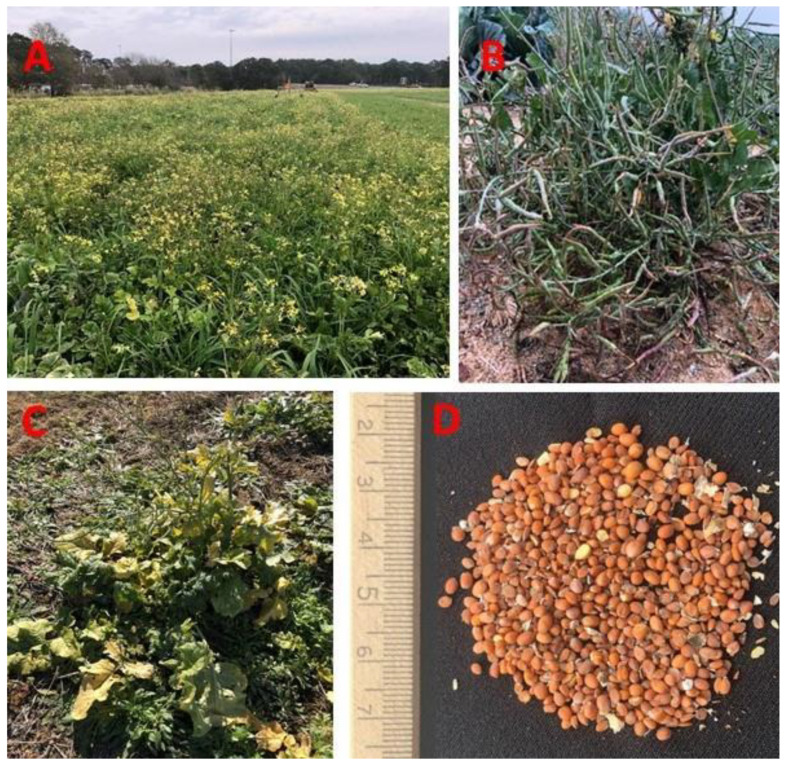
Wild radish (*Raphanus raphanistrum* L.) is a ubiquitous weed in southern Georgia. It can be seen growing profusely in and around the border of the fields (**A**). A single plant can produce a large number of pods (**B**), showing yellowing symptoms (**C**), and seeds were extracted from dried pods (siliques) (**D**).

Wild radish, *Raphanus raphanistrum* L., is a common weed found throughout the southeastern USA [1] (Figure 1A). It is also common in Canada, Australia, Kenya, and South Africa and is a weed of winter cereals, legumes, and vegetables [2,3,4,5]. Wild radish belongs to the family *Brassicaceae* and is classified as a winter annual (germinating in fall/dying in summer). However, it has been growing in the southeastern USA during the fall, winter, and spring in recent years. A single wild radish plant can produce between 1000 to 10,000 seeds depending on the time of year when germination occurs [6] (Figure 1B). Vast fields covered with wild radish are common in South Georgia, especially in the winter [7].

While surveying cucurbit fields infected with whitefly-transmitted viruses (WTVs) in the fall of 2020, wild radish growing in and around were found to exhibit virus-like symptoms. They included yellowing, interveinal chlorosis, and mosaic patterns (Figure 1C). Similar symptoms were observed on wild radish during the spring and winter seasons in 2020 and 2021. Cucurbit chlorotic yellows virus (CCYV) was identified in the symptomatic samples through high throughput sequencing (HTS) and confirmed by reverse-transcription PCR (RT-PCR). CCYV belongs to the genus *Crinivirus*, an emerging group of whitefly-transmitted viruses [8,9]. It was first discovered in Japan in 2004 as a disease that caused severe leaf yellowing in greenhouse-grown cucurbits and was named cucurbit chlorosis virus in 2004 [8,9,10]. Since then, it has been reported in Asia, Africa, and the Mediterranean regions of Europe [11,12,13], and from the USA recently [14,15,16,17]. CCYV is transmitted by the sweet potato whitefly, *Bemisia tabaci* (Gennadius, MED, and MEAM1 species complexes) in a semipersistent manner [8]. Criniviruses are responsible for worldwide losses of billions of dollars annually [18]. CCYV is reported to cause significant yield losses in Asian countries in cucumber and reduce the market value of melons by decreasing the sugar content [19,20].

In the southeastern USA, fall cucurbits are severely affected by CCYV and other WTVs [14,21,22]. These viruses are not reported to cause damage to winter vegetables, predominantly the members of Brassicaceae and spring crops that include watermelon. WTVs may not be a concern in winter and spring crops, but we have observed their incidence increases each year in fall-grown cucurbits in Georgia. Whitefly populations decline in Georgia in the winter months and start building up again when the weather gets warmer during summer [23]. Thus, we hypothesized that alternate hosts that harbor CCYV likely serve as reservoirs and aid in the rapid disease buildup in the field when the whitefly population increases each year. The potential of wild radish to serve as a reservoir host of CCYV was evaluated by: (1) assessing the ability of whiteflies to acquire the virus from wild radish and transmit it back to known cucurbit hosts of CCYV, and (2) determining if wild radish could serve as a viable feeding and reproductive host plant for the whitefly vector.

## 2. Materials and Methods

### 2.1. Source of Plants, Whiteflies, and Virus Culture

For the initial virus collection from host plants in the field, wild radish plant specimens were collected from the University of Georgia, College of Agricultural and Environmental Sciences Lang-Rigdon farm at Tifton (Lat. 31.515225, Long. −83.547716) and identified to species level based upon the identification keys [24] as well as descriptions maintained at https://blogs.cornell.edu/weedid/wild-radish/ (accessed on 15 February 2022). For whitefly evaluations in the fall of 2021, 2–3 expanded leaf stage wild radish seedlings were collected into pots from the same location for bioassaying *B. tabaci* for host suitability compared to known crop hosts in terms of adult feeding, oviposition, nymph development, and adult emergence. The whitefly host crops, cotton (*Gossypium hirsutum* L.) and squash (*Cucurbita pepo* L.), used for the whitefly bioassays, were grown from seed to the same 2–3 expanded leaf growth stage as the field-collected wild radish. All plants were reared from seeds for virus acquisition and transmission studies to ensure non-infection status prior to testing. Cotton, squash, and other cucurbit seeds were purchased from commercial seed companies (cotton from Deltapine, Bayer Crop Sciences, Whippany, NJ, USA; squash variety Gold Star from Seedway, Hall, NY, USA, and other cucurbits from Burpee Seeds, Philadelphia, PA, USA). Dried wild radish pods (siliques) were collected from the field, and seeds were extracted (Figure 1D) by forcing them open to increase germination percentage. All seedlings, including cotton, cucurbits, and wild radish, were planted in 2.7-inch plastic trays filled with potting medium (PRO-MIX, Quakertown, PA, USA).

*Bemisia tabaci* MED populations were reared on cotton, a good host for whiteflies and a non-host for CCYV. The culture of CCYV was maintained on commercial squash variety Gold Star (tolerant to powdery mildew) by periodic whitefly transmissions. The culture was always maintained inside cages (BugDorm, 160 µm aperture, MegaView Science Co., Ltd. Taichung, Taiwan) to avoid contamination with other viruses. All virus experiments were conducted in an insect-proof greenhouse at the University of Georgia Tifton Campus, maintained at a temperature of 28 ± 3 °C, 50 ± 20% relative humidity throughout the experiment. All whitefly bioassay experiments were conducted in the laboratory under a Jump Start Grow Light System (Greenhouse Megastore, Danville, IL, USA) at 24 ± 2 °C with 24:0 (L:D).

### 2.2. Detection of CCYV from High-Throughput Sequencing Data and RT-PCR

Leaves from a single wild radish plant exhibiting suspected virus symptoms of interveinal chlorosis collected in the fall of 2020 were shipped to Beijing Genomics Institute (BGI, San Jose, CA, USA) on dry ice for small RNA (sRNA) sequencing. Small RNA libraries were constructed and sequenced on a DNA Nanoball (DNB) small RNA sequencing platform, single-end read 1 × 50 bp (BGI, Hong Kong, China). Processing, assembly, virus detection, and identification from sRNA sequences were carried out using CLC Genomics Workbench 21 (CLC) (Qiagen, Redwood City, CA, USA). After removing adapter sequences and low-quality sequences, the reads were filtered on their size to retain reads of length between 18–30 nucleotides. Contigs were assembled de-novo from the reads following the parameters outlined by Pecman et al. [25]. These contigs were compared for similarity using BLASTn [26] against all sequences in a local viral database created from National Center for Biotechnology Information (NCBI) (http://www.ncbi.nlm.nih.gov/genome/viruses, accessed on 15 February 2022) with default parameters set in CLC. Sequences of RNA 1 and RNA 2 of CCYV assembled from sRNA sequences of wild radish were compared with those of the virus from cucurbit isolates in Georgia (MW629380.1 and MW629381.1). The nucleotide sequence identities were generated using BioEdit [27]. Finally, symptomatic wild radish samples collected from the field were tested for the presence of CCYV using RT-PCR (Table 1). The recipe and cycling conditions for cDNA preparation and PCR to detect CCYV are detailed in Kavalappara et al. [22].

### 2.3. Transmission of CCYV to Wild Radish and Back Transmission to Cucurbits

Adult whiteflies raised on cotton were provided 24 h of acquisition access period (AAP) on CCYV-infected squash. Viral infection on source plants (squash) was confirmed by RT-PCR before using them in transmissions as previously described [22]. After AAP, the whiteflies were provided an inoculation access period (IAP) of 24 h on wild radish plants. Three- to four-week-old wild radish seedlings with at least two completely developed true leaves were inoculated.

In the first set of experiments, a batch of ~500 whiteflies were released in whitefly-proof cages containing ten wild radish seedlings following the acquisition of the virus. In the second experiment, five wild radish plants were inoculated individually using clip cages. Groups of 30–50 whiteflies were tapped into clip cages after 24 h acquisition AAP and then attached to a recipient plant with whiteflies in contact with the abaxial surface of the leaf for inoculation of the virus [29]. Whiteflies were killed from the recipient plants after 24 h by treating with neonicotinoid insecticide ASSAIL^®^ 30SG (UPL NA Inc. King of Prussia, PA, USA) with the active ingredient acetamiprid at a rate of 0.025 g (a.i.)/100 mL water.

Back transmission of CCYV to cucurbit hosts was performed using clip cages with groups of 30–50 whiteflies. Five CCYV-infected wild radish plants were used as an inoculum source. Recipient plants consisted of five seedlings each of cantaloupe, yellow squash, green squash, and watermelon. All recipient plants were inoculated on the first true leaf on the adaxial side. After inoculation, all plants were kept in insect-proof cages in the greenhouse for 3 weeks and observed for symptom development. Samples were collected 21 days post-inoculation (dpi) to quantify the CCYV by RT-qPCR. Healthy plants exposed to virus-free whiteflies for 24 h and maintained under the same conditions in separate cages were used as controls.

### 2.4. Estimation of Copy Numbers of CCYV

RT-qPCR assays in a quantitative system using SSOAdvanced Universal SYBR Green Supermix (Bio-Rad, Hercules, CA, USA) were performed to quantify CCYV in wild radish and cucurbits. Cycle threshold (Ct) values from samples infected with CCYV were compared with a standard to estimate the copy number of viruses present in a sample. The standard consisted of a series of eight ten-fold dilutions of plasmid containing the fragment of CCYV in triplicate. DNA concentration in the plasmid was measured in ng/μL using Nanodrop (Thermo Scientific, Wilmington, DE, USA), and the number of copies was estimated based on the formula: the number of copies = (amount in ng × 6.022 × 10^23^)/(length of a vector in bp × 1 × 10^9^ × 650), in which the weight of a base pair (bp) is assumed to be 650 Da [30].

RNA was isolated from leaves of infected samples using Spectrum™ Plant Total RNA Kit (Sigma-Aldrich, St Louis, MO, USA) following the manufacturer’s instructions. cDNAs were prepared from 100 ng total RNA following the protocol described in Kavalappara et al. [22]. Five microliters of cDNA was used as template in the qPCR reaction of 25 µL, which also consisted of 12.5 µL of SSOAdvanced Universal SYBR Green Supermix and 1 µL each (10 µM) of forward and reverse primers. Details of primers used for RT-qPCR detection are given in Table 1. Each biological sample was tested in triplicate. Quantitative PCR was conducted using the CFX96 Touch Deep Well Real-Time PCR System^®^ (Bio-Rad, Hercules, CA, USA). An initial denaturation step (3 min at 95 °C) was followed by 40 cycles of denaturation (10 s at 95 °C) and a combined step of annealing and extension at 62 °C for 30 s. Melt curve analysis was conducted to evaluate the specificity of the fluorescence signal. The cycle thresholds and Melt curve were calculated by CFX Maestro Software (Bio-Rad).

### 2.5. Host Plant Suitability for Whiteflies

Three host plants, cotton, squash and wild radish, were grown to a 2–3 expanded leaf stage before beginning whitefly bioassays for host acceptability for feeding and oviposition and host suitability for nymph and adult reproduction. Both cotton and squash were reared from seed in E-36L2 Percival Growth Chambers at 28 ± 2 °C, 14:10 (L:D) to a 2–3 leaf stage. The wild radish was collected from the field in the fall of 2021 to best simulate its plant condition when whiteflies are moving out of cotton and vegetables in the field [23].

For the host acceptability for feeding and oviposition study, standard 2.5 cm diameter clip cages [31] were placed on the top expanded leaf of the host plants with approximately 15 male–female pairs of whitefly adults of mixed ages in each cage. After 48 h, adults were collected and counted as either live or dead (all dead would indicate a non-acceptable feeding host plant resulting in significant adult mortality). The plants were held for 1 week when the caged leaves were collected, and all whitefly eggs and nymphs were counted. The test was repeated using 10 replicates in a complete randomized design.

For the host suitability for nymph and adult reproduction test, the three individual host plants were potted in small vases (10 cm diam.) and enclosed with clear plastic bioassay tube cages (9 cm diam., vol = 1.9 L) covered with chiffon fabric. Thirty newly emerged, 1-day old whitefly adults (approximately 50% female) were added into the cages. Live adults, eggs, and nymphs were counted after 1 week and the remaining adults were then removed. Newly emerged whitefly adults were estimated 2 weeks later, from the cages daily until all emergence ceased. The total number of adults produced per 30 live adults after one-week oviposition is a crude net reproduction estimate, with >30 value indicating an increasing population based on ~one-tenth of the potential oviposition [32]. The total oviposition, nymph, and adult data were subjected to individual analysis of variance by using Proc GLM in SAS (SAS Institute, Raleigh, NC, USA) using a randomized complete block plot design. Significant treatments were reported and means graphed with standard errors for describing host plant effects. The LSD test was used for non-repeated measures data such as one-time egg counts (*p* < 0.05) following a significant treatment effect (*p* < 0.05).

## 3. Results

### 3.1. Detection of Cucurbit Chlorotic Yellows Virus on Field Samples and Its Characteristics

**Figure 2:**
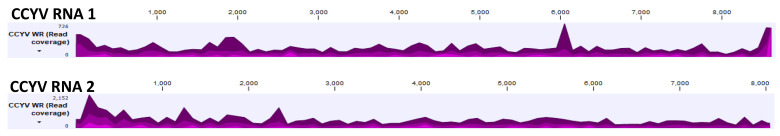
Read coverage maps of cucurbit chlorotic yellows virus (CCYV) detected by high throughput sequencing of small RNAs of symptomatic wild radish. Scaled genome positions of the virus are shown above the histograms and the Y-axis represents the coverage in the number of reads. Within the specified aggregation bucket from top to bottom, the colors mean: the maximum coverage value (read count), the average coverage value, and the minimum coverage value.

Sequences of total sRNA were generated from leaves of a single wild radish exhibiting interveinal chlorosis. The plant was collected from a cucumber field infected with WTVs, including CCYV. A total of 15,026,252 reads were generated from the sample, of which 14,521,505 reads were retained after removing adapters and reads of inferior quality. Ninety-four contigs with a minimum size of 150 nt were assembled from the sequences, out of which eleven contigs belonged to CCYV. One contig each of petunia vein clearing virus (PVCV) and citrus endogenous pararetrovirus (CitPRV) were also detected. Sequences of other WTVs virus present in the region, including closely related crinivirus cucurbit yellow stunting disorder virus (CYSDV) were not detected from this sample. The number of sRNAs mapping to genomes of PVCV and CitPRV were very low and hence we ruled out their presence. In contrast, CCYV specific small RNAs were distributed throughout the genome with 93,141 and 37,437 reads mapping to RNA 1 and RNA 2 of the reference sequence of CCYV, respectively (NC_018173 and NC_018174) (Figure 2).

The near-complete sequence of RNA 1 and RNA 2 of CCYV could be assembled from sRNA (GenBank Accession numbers: OM489400, OM489401). The sequences of RNA 1 and RNA 2 of CCYV from wild radish shared 99.90% and 99.95% nt sequence identity with respective RNAs of the cucurbit isolate of CCYV (MW629380.1 and MW629381.1) reported earlier from Georgia.

Apart from the single plant tested for the presence of viruses, more wild radish plants collected from in and around vegetable fields were tested for the presence of CCYV using RT-PCR. CCYV was detected in five out of 50 samples collected during the winter of 2020 and eight out of 81 samples collected during the spring of 2021.

### 3.2. Transmission of Cucurbit Chlorotic Yellows Virus to Wild Radish

**Figure 3:**
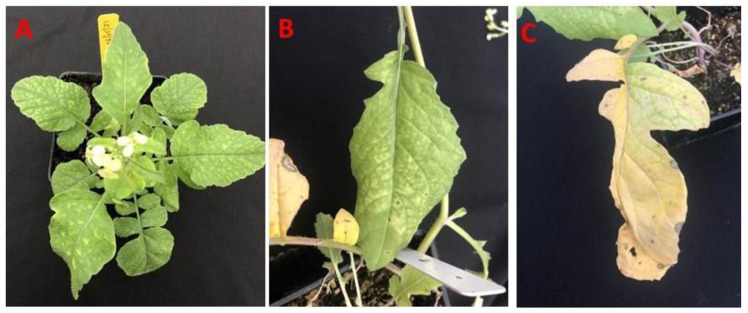
Symptoms induced by cucurbit chlorotic yellows virus on wild radish (*Raphanus raphanistrum):* mild yellowing at 2 weeks after inoculation (**A**); interveinal chlorosis (**B**); late symptoms yellowing and drying of lower leaves (**C**).

To assess the potential of wild radish to serve as a reservoir of CCYV, transmission assays to wild radish were conducted. In mass inoculations, ~500 whiteflies after 24 h AAP on CCYV infected squash were released onto ten wild radish seedlings in cages. Mild yellowing symptoms were observed on all ten seedlings of wild radish by 2 weeks (Figure 3A), which later turned into clear interveinal chlorosis in some plants (Figure 3B) and yellowing in late stage (Figure 3C). Control plants that were inoculated with non-viruliferous whiteflies did not produce any symptoms. CCYV was detected from seven out of ten plants (70% infection) exposed to viruliferous whiteflies but not from any of the control plants.

When wild radish was exposed to viruliferous whiteflies carrying CCYV in clip cages, all five plants developed symptoms of mild yellowing that later turned into severe interveinal chlorosis within 3 weeks after inoculation. CCYV was detected by RT-qPCR in samples collected 21 days after being exposed to viruliferous whiteflies from all five plants but not on control plants (100% infection) (Table 2).

### 3.3. Back Transmission of CCYV from Wild Radish to Known Cucurbit Hosts

To examine the potential of CCYV spread from wild radish to crops, the transmission of CCYV from infected wild radish back to cucurbit hosts was performed. Clip cages containing 30–50 whiteflies after 24 h AAP were attached to five seedlings, each of three species of cucurbits that are highly susceptible to CCYV, including yellow squash, green squash, watermelon, and cantaloupe. All five plants in each of the three species of cucurbits started exhibiting symptoms of yellowing after 2 weeks of inoculation. By the end of 3 weeks, all plants exposed to viruliferous whiteflies exhibited interveinal chlorosis, while control plants exposed to nonviruliferous whiteflies did not develop any symptoms. CCYV was detected from each plant of all three species inoculated by RT-qPCR in samples collected 21 days after inoculation (100% infection) (Table 2).

**Figure 4:**
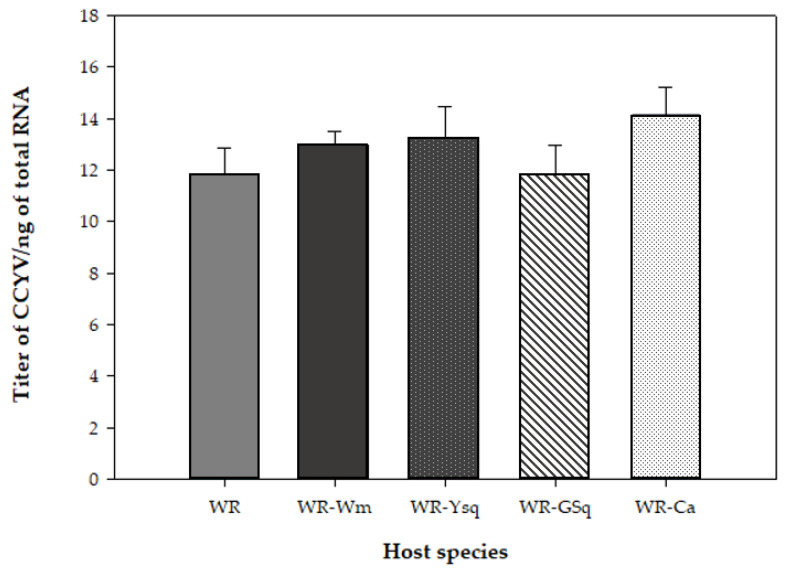
Estimate of cucurbit chlorotic yellows virus (CCYV) titer in wild radish (WR) and cucurbits hosts after back transmission from wild radish (WR-Wm: Watermelon, WR-Ysq: yellow squash, WR-GSq: green squash, WR-Ca: cantaloupe) by reverse transcriptase-quantitative polymerase chain reaction (RT-qPCR) using primers detailed in Table 1. Data points represent the mean estimated copy numbers of CCYV from five different plants of each species. Each plant was replicated three times. Bars indicate the standard error of the mean.

The copy numbers of CCYV on wild radish and other cucurbit hosts were estimated and compared (Figure 4). Wild radish supported a high load of CCYV comparable to that of known cucurbit hosts.

### 3.4. Host Plant Suitability for Whiteflies

**Figure 5:**
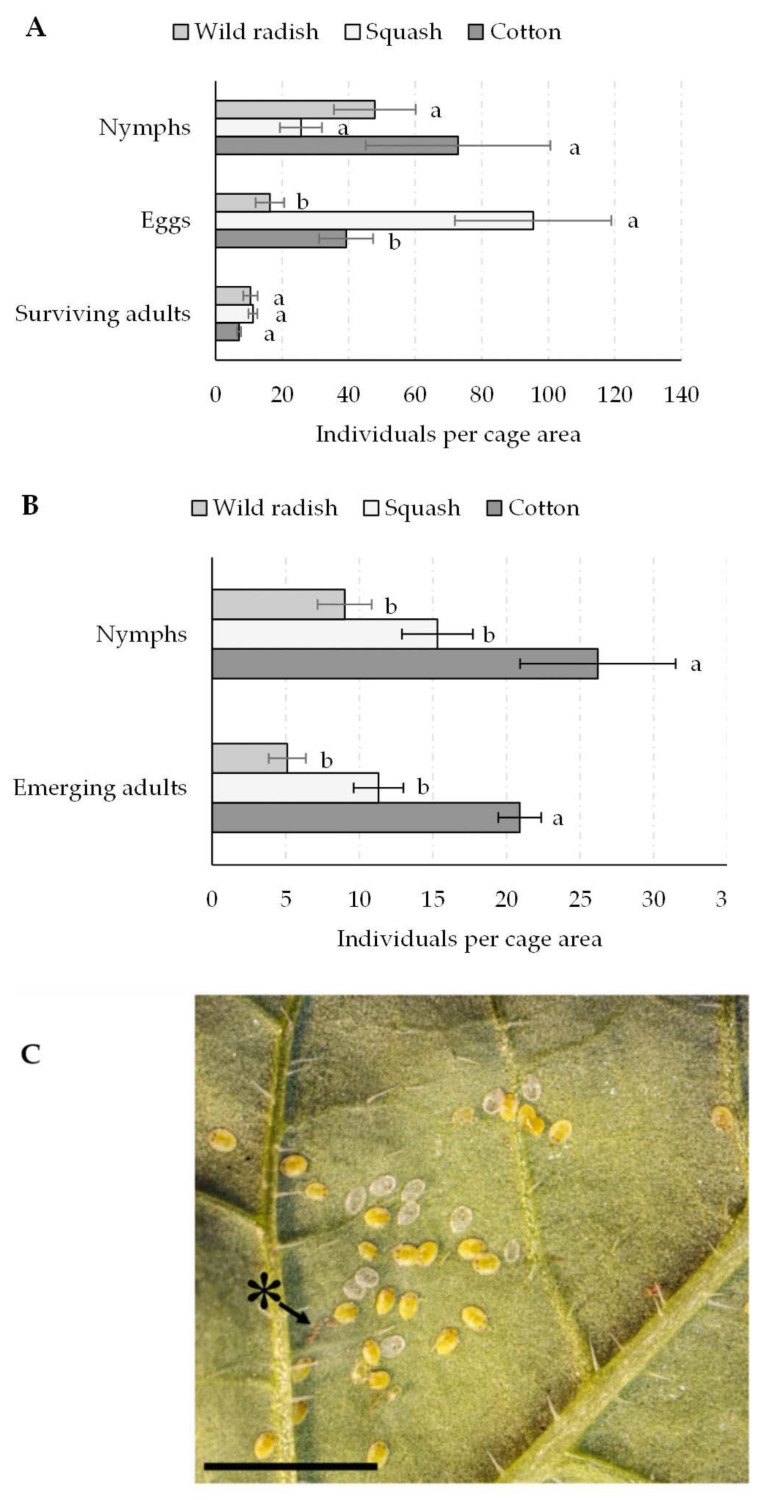
Whitefly numbers by stage on wild radish, squash, and cotton after 48 h in a clip cage on the top expanded leaf (**A**) and nymphs and adults produced after 1 and 2 weeks, respectively, in a tube cage with a plant with two expanded leaves (**B**) The statistical difference is represented as ‘a’ and ‘b’. Different immature stages of *Bemisia tabaci* and exuviae left from emerged adults on *Raphanus raphanistrum* leaf abaxial surface. * = eggs; bar = 5 mm (**C**).

Wild radish appeared to be a relatively acceptable host plant to whiteflies collected from cotton in terms of adult survival after feeding for 48 h (Figure 5A) and the production of new adults after developing on the plant (Figure 5B). Even though squash was a preferred host for oviposition, wild radish did not differ significantly from cotton in surviving adults after 48 h feeding, eggs laid, or resulting nymphs after 1 week (Figure 5A,C). Whether or not wild radish could sustain a whitefly population over time, the preliminary reproduction data (Figure 5B) suggest that, although not as good as the colony host plant, cotton, the nymphs, and emerging adults were not significantly different from squash. The number of adults produced per 30 adults on wild radish (5.1 ± 1.3) was half that for squash (11.3 ± 1.7), but not significantly different. However, both differ from cotton (20.9 ± 1.5), suggesting that wild radish was not as good a reproductive host plant as the base colony crop but could potentially supply a small population if a preferred host is unavailable.

## 4. Discussion

Cucurbit chlorotic yellows virus (CCYV) belongs to the genus *Crinivirus*, an emerging complex of primarily whitefly transmitted viruses (WTVs) associated with cucurbit yellows disease. In the last few years, a heavy incidence of whiteflies and viruses transmitted by them, including CCYV has been observed in the southeastern USA in the fall. CCYV and other WTVs are believed to survive on alternate hosts between the two main crop seasons. Wild radish is a ubiquitous weed found in the southern USA and many countries around the world. It is found germinating annually throughout the year in the region and has been widespread in fields and along the borders in recent years.

CCYV was detected in field samples of wild radish in 2020 and 2021, indicating that it is established in wild radish in the region. However, the presence of symptoms did not always correlate with infection by CCYV. This could be due to the presence of other viruses, including CYSDV, with which CCYV shares the same vectors [22]. CCYV derived sRNAs were abundant in the total sRNA reads from wild radish. The near-complete nucleotide sequence of CCYV could be retrieved from the sRNA sequences with a high degree of confidence. When compared with isolates of CCYV infecting cucurbits from the region, the nucleotide sequence of the CCYV from wild radish was 99.9% identical. This demonstrates that the isolate of CCYV infecting wild radish is the same as those infecting cucurbits in Georgia. Sequences of another closely related crinivirus, CYSDV was not detected from this sample. One contig (~150 nucleotides) each of PVCV and CitPRV were detected in HTS, but further analysis ruled out their presence in the samples.

When assessing the potential of wild radish to serve as a reservoir of CCYV, transmission assays of CCYV were conducted to wild radish under controlled greenhouse conditions. Wild radish became infected with CCYV and developed symptoms associated with CCYV infection, which was further confirmed by detecting of the virus with RT-qPCR. To examine the potential of CCYV spread from wild radish to cucurbit crops, the transmission of CCYV from infected wild radish back to cucurbit hosts was performed. CCYV was efficiently transmitted to three species of cucurbits which are highly susceptible to CCYV, including squash, watermelon, cucumber, and cantaloupe, and developed typical symptoms of CCYV.

Further, the titer of CCYV in wild radish was on par with that of cucurbits included in the study. It has been suggested that a certain threshold level of virus is required for efficient acquisition and transmission of the virus from a plant species [33]. This hypothetical threshold has been found to be host-dependent in the case of tomato yellow leaf curl virus (TYLCV) [34]. The amount of CCYV accumulated in wild radish is sufficient for efficient acquisition and successful transmission of the virus to recipient hosts. Consequently, wild radish is likely an excellent reservoir for virus survival when crop reservoirs are not present.

The crucial role played by weeds in virus spread and overwintering has been well documented [35,36,37,38,39,40,41,42,43,44]. In the case of viruses not transmitted by seed, such as criniviruses, weeds are especially important for survival between crop seasons [45,46,47]. Natural infection of CCYV has been observed mainly on cucurbits, although species belonging to Asteraceae, Chenopodiaceae, Convolvulaceae, and Solanaceae were also found to be susceptible under experimental conditions [8]. CCYV was detected from two weeds of the family *Brassicaceae*, *Capsella bursa-pastoris,* and *Sisymbrium* sp., along with several other weeds [46].

This study illustrates that wild radish is an excellent reservoir from which whiteflies can acquire and transmit CCYV to cultivated cucurbits. The ability of wild radish to persist through the winter, and its abundance, make it potentially important reservoirs for CCYV when crop reservoirs are not present. Whitefly populations start building up in spring and can be exceptionally high throughout the southeastern USA in the fall season. Even a few infected wild radish plants can serve as foci from which whiteflies can acquire the virus and spread in the field quickly. CCYV is often found in co-infections with another crinivirus, CYSDV which induces similar symptoms and shares the same vectors. Initial attempts to transmit CYSDV to wild radish were unsuccessful, however, and further studies are required to establish this.

This is also the first study that demonstrates the ability of a member family *Brassicaceae* to serve as an excellent host for CCYV. The potential role of brassica crops to serve as overwintering hosts of CCYV and other criniviruses in the southeastern USA and other parts of the world needs to be further studied.

## Figures and Tables

**Table 1:** Primers used to detect cucurbit chlorotic yellows virus (CCYV) in this study.

Assay	Primer Name	Sequence 5′-3′	Tm (°C)	Amplicon Size	References
RT-PCR	CCYV-RDRP-1515F	CTCCGATAGATCATCCCAAATC	62	953	[15]
	CCYV-RDRP-1515R	TCACCAGAAACTCCACAATCTC			
RT-qPCR	CCYV-F	GGTTTACACACCCGGTGAGTTT	62	91	[28]
	CCYV-R	TGAAATTAGGGCTTGCTTCCA			

**Table 2:** Infection of wild radish with cucurbit chlorotic yellows virus (CCYV) and back transmission to cucurbit hosts. Wild radish infected with CCYV were used as source plants for back transmissions to cucurbit hosts. Virus infections on inoculated plants were determined by RT-qPCR 3 weeks after inoculation.

	Inoculated Plant Species	Symptoms ^1^	RT-qPCR Detection (No of Plants Positive/Inoculated)
Transmissions to wild radish	Wild radish; *Raphanus raphanistrum* (Mass exposure)	IC, Y	7/10
	Wild radish; *R. raphanistrum* (Clip cage inoculation)	IC, Y	5/5
Back transmissions to cucurbit hosts of CCYV	Yellow squash; *Cucurbita pepo*	IC, Y	5/5
	Green squash; *C. pepo*	IC, Y	5/5
	Watermelon; *Citrullus lanatus*	IC, Y	5/5
	Cantaloupe; *Cucumis melo var. cantalupensis*	IC, Y	5/5

^1^ Abbreviations used for symptoms: IC: Interveinal chlorosis; Y: yellowing.

## Data Availability

Not applicable.
